# Functional near-infrared spectroscopy neurofeedback of dorsolateral prefrontal cortex enhances human spatial working memory

**DOI:** 10.1117/1.NPh.10.2.025011

**Published:** 2023-06-01

**Authors:** Keshuang Li, Jinhao Yang, Benjamin Becker, Xianchun Li

**Affiliations:** a East China Normal University, School of Psychology and Cognitive Science, Affiliated Mental Health Center, Shanghai Key Laboratory of Mental Health and Psychological Crisis Intervention, Shanghai, China; b University of Electronic Science and Technology of China, The Clinical Hospital of Chengdu Brain Science Institute, MOE Key Laboratory for Neuroinformation, Chengdu, China

**Keywords:** spatial working memory, neurofeedback, functional near-infrared spectroscopy, neuromodulation, dorsolateral prefrontal cortex, attention

## Abstract

**Significance:**

Spatial working memory (SWM) is essential for daily life and deficits in this domain represent a common impairment across aging and several mental disorders. Impaired SWM has been closely linked to dysregulations in dorsolateral prefrontal cortex (DLPFC) activation.

**Aim:**

The present study evaluates the feasibility and maintenance of functional near-infrared spectroscopy neurofeedback (fNIRS-NF) training of the DLPFC to enhance SWM in healthy individuals using a real-time fNIRS-NF platform developed by the authors.

**Approach:**

We used a randomized sham-controlled between-subject fNIRS-NF design with 60 healthy subjects as a sample. Training-induced changes in the DLPFC, SWM, and attention performance served as primary outcomes.

**Results:**

Feedback from the target channel significantly increased regional-specific DLPFC activation over the fNIRS-NF training compared to sham NF. A significant group difference in NF-induced frontoparietal connectivity was observed. Compared to the control group, the experimental group demonstrated significantly improved SWM and attention performance that were maintained for 1 week. Furthermore, a mediation analysis demonstrated that increased DLPFC activation mediated the effects of fNIRS-NF treatment on better SWM performance.

**Conclusions:**

The present results demonstrated that successful self-regulation of DLPFC activation may represent a long-lasting intervention to improve human SWM and has the potential for further applications.

## 1 Introduction

Working memory (WM) represents a core executive function and is essential for an entire range of cognitive domains, including planning, skill learning, and problem-solving. The WM refers to a limited functional system that can hold, update, and monitor a strictly limited amount of verbal and visuospatial information.^1^ Previous studies have found a greater age-related decline in spatial WM (SWM) compared to verbal WM,^2^ and SWM was demonstrated to be selectively impaired in several mental disorders, such as schizophrenia,^3^ attention-deficit/hyperactivity disorder,^4^^,^^5^ major depression,^6^^,^^7^ post-traumatic stress disorder,^8^ and anxiety disorder.^9^ Together, SWM is not only essential for daily life but may also represent a transdiagnostic impairment across aging and several mental disorders.

It has been advocated that the active maintenance of location-specific representations in SWM is mediated by focal shifts of selective attention to the memorized location, and if focused attention is forced to orient away from the memorized location, WM accuracy will decline.^10^^,^^11^ A long-standing suggestion proposes that age-related SWM impairments are consistent with a greater degree of age-related slowing observed in visuospatial (as compared with verbal) attention.^12^ Similar deficits have been consistently reported in psychiatric disorders characterized by impaired SWM. For instance, anxiety selectively disrupts the efficient allocation of attention in spatial tasks while sparing verbal task performance.^13^^–^^17^ In anxiety disorders, biased attention in the service of anxiety-relevant goals has been reported and may lead to fewer resources being available to support SWM performance.^17^ Impairments in SWM were significantly associated with attention control abilities that are known to be impaired in normal aging and disease states, thus it is critical for improving SWM to pay more attention to the common neural basis of SWM and attention processes.

Neurofeedback (NF) techniques have been a promising non-invasive means to improve cognitive control by regulating associated neural activity.^18^^,^^19^ The corresponding methods employ a biofeedback approach that uses real-time information about brain activity to enable self-regulation of a particular neural signal.^18^^,^^20^ Functional near-infrared spectroscopy (fNIRS) imaging has advantages over other hemodynamic imaging techniques in terms of its relatively high and acceptable spatial resolution, lower costs, and fewer contraindications, as well as robustness against motion and susceptibility to artifacts.^21^ A growing number of studies have thus begun to utilize fNIRS-based NF training (fNIRS-NF), which may represent a promising strategy to achieve self-regulation over regional brain activity.^22^ Some studies demonstrated the feasibility to gain regulatory control over prefrontal brain activity via fNIRS-NF and its potential to enhance associated executive functions, including WM in the letter N-back task.^23^^,^^24^ In previous work, we demonstrated that healthy human subjects can learn volitional and regional-specific control over the lateral prefrontal cortex which in turn enhanced cognitive flexibility in the attentional set-shifting task.^25^ A recent study demonstrated the feasibility of fNIRS-NF to improve human spatial memory but not WM by manipulating the neural activity of the lateral parietal cortex.^26^ Given the important associations between SWM dysfunction and cognitive impairment in normal aging and psychiatric disorders, determining the potential of fNIRS-NF to enhance SWM could represent a promising strategy for novel interventions. Furthermore, it is important to determine whether the training effects of fNIRS-NF can be maintained over longer periods beyond the initial training period. This is in particular an important step for a potential clinical application. Although several previous studies have reported that the behavioral effects induced by fNIRS-NF could persist for weeks or even months after intervention,^27^^–^^30^ these studies included long training periods (3 to 12 training sessions). Thus, the special time course of long-term effects induced by a single training session has been characterized by follow-up measurement in the present study, which will further verify the feasibility of fNIRS-NF.

SWM and attention processes are known to be subserved by overlapping neural substrates, including lateral prefrontal regions together with parietal regions.^9^^,^^31^^,^^32^ Visuospatial processing or spatial location processing depends on the dorsal stream, consisting of the posterior parietal cortex (PPC) and projection to the dorsolateral prefrontal cortex (DLPFC).^33^ Importantly, human imaging studies demonstrated a prototypical hemispheric functional segregation pattern, such that spatial stimuli primarily recruited a right-hemispheric network.^16^ Electroencephalogram studies have revealed that cross-frequency phase synchronization between theta and gamma oscillations at PPC is associated with the successful maintenance of relevant objects in SWM, and alpha activity amplitude is related to the efficient suppression of irrelevant information.^34^ In contrast to PPC, which is associated with the passive storage of spatial information related to WM load, the DLPFC has been implicated as a critical area for higher-level executive processing like updating information and suppression of distraction. Previous transcranial magnetic stimulation studies showed that stimulation on the right DLPFC enhanced the DLPFC function within the central executive system at the top-down attentional level and improved SWM capacity, particularly when task difficulty demands more complex mental manipulations.^33^ The contribution of the DLPFC in the network of SWM has additionally been confirmed by normal aging and disease states studies. DLPFC oxygenation is correlated with better visuospatial working memory performance, e.g., older adults showed less activity in the DLPFC when they performed comparably to younger people.^35^ Likewise, SWM deficits in psychiatric disorders are associated with aberrant DLPFC activation.^36^^–^^38^ These findings suggested that increasing DLPFC activity may represent a promising strategy to improve SWM performance. Previous studies suggested that hemodynamic responses (HRs) in the PFC related to WM load can be robustly assessed by NIRS;^39^ thus, the present study developed and evaluated an fNIRS-based NF training that targets upregulating the right DLPFC to enhance SWM in humans. In line with the neural network in SWM, neuromodulating the activity of this region may also have potential effects on the PPC activity. To control for unspecific effects other than training, we used a randomized sham-controlled between-subject experiment design with a total of n=60 health college participants as the sample. Based on previous studies,^25^^,^^40^^,^^41^ we expected that participants in the experimental but not the control group would learn to successfully upregulate regional-specific activity in the right DLPFC and that this would be accompanied by behavioral-level changes in SWM performance.

## 2 Methods and Materials

### 2.1 Participants

Sample sizes for the main analytic approaches were calculated using G*Power version 3.1, based on the recent study suggesting that if the goal of a NF study is to show that individuals can improve their behavior compared to baseline, effect sizes may be large.^42^ We calculated the sample size required for a repeated measures analysis of variance (ANOVA) with two groups and two behavioral task time-points to detect effects with large to very large effect sizes. Thus, assuming an ES of 0.4 and a predefined α of 0.05, a power of at least 0.80 results in a total sample size of 52, or 26 per group. Therefore, a total of n=60 healthy young students were enrolled in the present study. To control for the unspecific effects of the training procedures on the primary outcomes, the NF training was embedded in a randomized, sham-controlled between-subject experimental design. Participants were randomly assigned (30 participants in each group) to receive either real-time feedback (the experimental group) or sham feedback (the control group) during the training. Participants were randomized without stratifying for further variables. All participants provided written informed consent. The study had full ethical approval from the local ethics committee of East China Normal University.

### 2.2 Experimental Protocols and Procedures

**Fig. 1:**
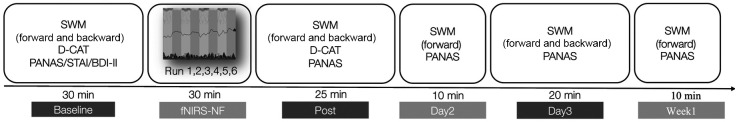
Experimental procedures for the six experimental sessions.

Participants were scheduled for six experimental sessions (as shown in Fig. 1), including one baseline session (referred to as Baseline), and one fNIRS-NF training session (referred to as fNIRS-NF), as well as four follow-up sessions (referred to as post, day2, day3, and week1). Baseline, fNIRS-NF, and post-training sessions were conducted within 1 day, and day2 and day3-training sessions were conducted 1 and 2 days after the fNIRS-NF training, respectively. The final assessment was scheduled 1 week after the fNIRS-NF training (week1). During all sessions, participants were administered an SWM task,^43^ including forward and backward versions of the task. Specifically, participants were administered the forward task in all sessions while the backward task was administered at baseline, post, and day3-training sessions. Previous studies demonstrated that the backward visuospatial span task was more difficult and involved greater WM demands than the forward task.^44^^,^^45^ Thus, we initially considered that a single training session might be more likely to improve the forward task, and also to avoid repetition-associated practice effects for the behavioral task, only the forward task was included in day2 and week1-training sessions. During baseline and post-training sessions, participants were also administered the digit cancellation test (D-CAT).^46^ The D-CAT aims to evaluate focused attention and selective attention, which are associated with SWM.

The State Anxiety Inventory^47^ and the Beck depression inventory-II (BDI II)^48^ were administered during the baseline training session to control for potential confounding effects of baseline between-group differences in psychopathological symptom load, while the positive and negative affect schedule (PANAS)^49^ was administered on each experimental day to control for nonspecific effects of training on mood. To further control potential effects between the real and the sham training group, all participants were required to rate their training success (scale ranging from −4 to 4) and self-report the experimental condition (real feedback or sham feedback).

### 2.3 Neurofeedback Training Protocols, NIRS Data Acquisition, and FEEDBACK

**Fig. 2:**
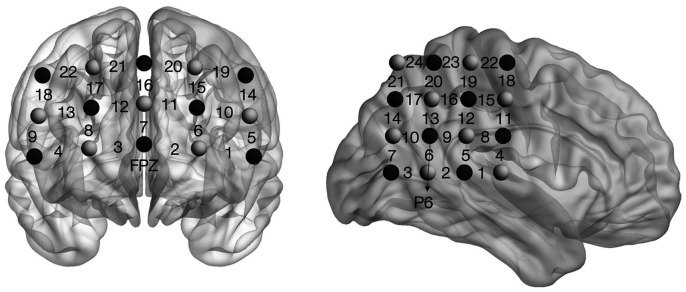
One 3*5 probe patch (3-cm distance between the emitter and detector) was placed over the prefrontal regions of each participant. The middle optode of the lowest row of the patch was placed on the frontal pole midline point (FPz in the International 10 to 20 system, as the reference site). The middle column of the probe was aligned along the sagittal reference plane. The correspondence between the fNIRS channels and the measurement points was referred to in the positioning template provided by Jichi University^50^ (as shown in Table S1 in the Supplementary Material). The other 4*4 probe patch was placed over the rTPJ of each participant, with the optode placed on P6, according to the International 10 to 20 system. The row of the probe was aligned along the sagittal reference plane. The correspondence between the fNIRS channels and the measurement points on the cerebral cortex was displayed based on the results of the virtual registration method, which had been confirmed by a multi-subject study of anatomical craniocerebral correlation,^51^ as shown in Table S2 in the Supplementary Material.

The fNIRS-NF training session included six runs of alternating rest and regulation blocks (four blocks per run, block duration being 25 s). The experimental group received real-time feedback from fNIRS channel 9 located over the right DLPFC, whereas the control group received feedback from one random participant who had previously undergone the experimental training (yoke feedback). The HR signals were assessed using an fNIRS System (ETG-7100, Hitachi Medical Corporation, Tokyo, Japan) at a sampling rate of 10 Hz. Locations of the optodes and feedback channel are displayed in Fig. 2. To accustom the participants to the equipment and to reduce variance related to trial-and-error attempts during the initial training run, all participants received real-time feedback in run1 and were required to explore suitable regulation strategies during the subsequent four regulate blocks. Participants were told that they could employ the strategies they discovered during the run1 or continue to find new strategies to increase brain activity during the subsequent training.

In line with previous fNIRS-NF studies, the feedback was based on the oxy-Hb signal.^26^^,^^52^^,^^53^ Oxy-Hb signals were acquired by the ETG-7100 system, and online receiving, preprocessing, and real-time feedback of the oxy-Hb signal were computed using a novel real-time fNIRS-NF platform developed by the authors (MindGym, MG®, Patent No. CN202110959972.3). Specifically, to reduce the physiological noise caused by heartbeat, respiration, and other physiological processes, the raw oxy-Hb signal was smoothed using a 2 s moving average window. A baseline was calculated by taking the average of signals 10 s before the first block and was subsequently subtracted from the smoothed signal. The feedback signal was computed in real-time from the signal of the a priori target channel 9.

The online feedback was visually displayed to the participants in a similar way to a time series (see Fig. 1). The height of the line visualized the neural activity level in the chosen feedback channel 9 (the higher the height of the line, the higher the activity). Participants were asked to raise the line height as high as possible by regulating their brain activity by using mental strategies. We decided to embed the feedback in this way to help participants compare the training effects of different regulate blocks and increase training success. The conditions (rest/regulate) were visually presented to the participant via two background colors (see Fig. 1). The rest condition was indicated by a rainy background, and the regulate condition was indicated by a sunny background.

### 2.4 Instructions for the Participants

Participants were informed that the purpose of the fNIRS-NF training was to examine whether they could learn to upregulate their brain activity. Given that explicit strategy instruction is not necessary for successful NF-assisted acquisition of neural regulation,^54^ no explicit strategies for regulation were provided to the participants. Participants were instructed not to control the height of the line by physical means, such as breathing or head/body motion but rather to discover efficient mental control strategies.

### 2.5 Behavioral Assessments

To assess whether fNIRS-NF of the right DLPFC leads to generalized improvements in cognitive control, participants were administered the SWM task and the D-CAT.

During the SWM task, participants were presented with sequences of visuospatial stimuli of varying lengths and repeated sequences of stimuli in the same (forward) or reversed (backward) order. The stimuli consisted of squares appearing at 25 different locations and were pseudo-randomized into 24 sequences. The sequence length varied between 3 and 10 stimuli and 3 sequences of each length in both the forward and backward tasks. Thus, the forward or backward task has 24 trials each in a single run, with each run lasting ∼5  mins. Each cross was visible in the matrix for 2000 ms. The inter-stimulus interval was 800 ms, during which the matrix was empty. Each time a sequence ended; the participants repeated the squares by clicking on the correct locations with the mouse. In both tasks, the participants did not know the sequence length beforehand. The dependent variables were the total number of correctly recalled location sequences.^43^ The order of stimulus length was randomized between the times of tasks.

The D-CAT sheet consisted of 12 rows of 50 digits, each containing 5 sets of the numbers 0 to 9 arranged in random order. Participants were instructed to search for target number(s) [the first involving a single target number (6); a second with two target numbers (9 and 4); and a third with three (8, 3, 7)] with a slash mark as quickly and as accurately as possible. During the second and third trials, it was stressed that all of the target numbers should be canceled without omission.^46^ Each test sheet had a time limit of 60 s, and the sheets were presented one right after the other. The order of digits was randomized between the times of tasks.

### 2.6 Offline Preprocessing and Analyses

The fNIRS raw data were preprocessed and analyzed using the NIRS toolbox in statistical parametric mapping (SPM)^55^^,^^56^ and in-house scripts in MATLAB (The MathWorks, Inc.). During preprocessing, an NIRS analysis package was applied to correct head motion during training, and a second-order detrending was applied to remove the baseline drifts and low-pass filtering [Gaussian smoothing with full width at half maximum (4s)] was employed to remove high-frequency noise. A generalized linear model (GLM) approach was employed to model the task-related HR on the individual level, including the regulation periods modeled by a boxcar function while the rest periods were included as an implicit baseline. Serial autocorrelations in the GLM analysis were accounted for by employing pre-whitening as incorporated in NIRS SPM [AR (1) model, in line with recommendations by Huppert^57^].

For the group-level analyses, beta estimates were obtained for each participant and channel. The primary outcome to determine the training success on the neural level was oxy-Hb change over the training runs in the target channel (right DLPFC; channel 9). To further control the unspecific effects of training or effects of mental effort on DLPFC activity, individual-level beta values from the target channel were subjected to group-level activation analyses comparing the experimental and the control group. Differences were considered significant using a channel-level threshold of p<0.05 (Bonferroni-corrected).

### 2.7 Evaluation of Training Success and Primary Outcomes

Training-induced oxy-Hb changes in the DLPFC target channel served as the primary outcome to evaluate the training success on the neural level. First, channel-specific activity beta estimates were employed as dependent variables, and the effects of training were initially determined by employing mixed ANOVA models, including the between-subject factor Group (real feedback versus sham feedback) and the within-subject factor training Time (run2/run3/run4/run5/run6). Significant effects were explored by employing appropriate Bonferroni-corrected post hoc tests. Then channel-specific activity beta estimates were employed as dependent variables again, and the effects of training were further determined by employing one-way ANOVA models in each group including the within-subject factor training Time (run1/run2/run3/run4/run5/run6). Significant effects were also explored by employing appropriate Bonferroni-corrected post hoc tests.

The total number of correctly recalled location sequences in the SWM task was employed as dependent variables, and the effects of training on the behavioral level were determined by employing mixed ANOVA models, including the between-subject factor Group (real feedback versus sham feedback) and the within-subject factor time (baseline/post/day2/day3/week1). Significant effects were further explored by employing appropriate Bonferroni-corrected post hoc tests. The total performance in the D-CAT task was used as the dependent variable in mixed ANOVA models which included a between-subject factor of group (real feedback versus sham feedback) and a within-subject factor of time (baseline versus post). These mixed ANOVAs were performed to test the effects of training on the attention performance. Associations between neural and behavioral training success were explored by employing analyzing correlations between training-induced DLPFC oxy-Hb changes and behavioral indices in the experimental group. Statistical analyses were carried out using SPSS version 22.0 (IBM, Inc.).

### 2.8 Up-regulation Strategies

After the training, all participants were asked to report the strategies they employed during the training. The reported strategies were qualitatively assessed by five independent raters (two males). To control for different strategies between the training groups, the frequencies of the reported regulation strategies were compared between the experimental group and the control group using the Pearson $X^{2}$ test.^58^

## 3 Results

### 3.1 Data Quality Control

While 55 out of the 60 participants completed the whole experiment procedure, initial examination of the primary neural outcome (oxy-Hb) and behavioral outcome (total number of correctly recalled locations in SWM task) data quality identified 3 participants as outliers (outliers were defined as subjects that deviated larger than 2 standard deviations from the mean, outliers were additionally confirmed using the SPSS outlier detection function). Consequently, data from these participants were excluded from all analyses resulting in a total of n=52 participants for the primary analysis (n=27, experimental group; n=25, control group).

### 3.2 Assessment of Confounders

The demographic information and psychopathological load of all participants were reported in Table 1. A mixed two-way ANOVA with the factors time (baseline/post/day2/day3/week1) and group (real feedback versus sham feedback) and the dependent variable self-rated mood levels revealed a main effect of time ($F_{\mathrm{PANAS}\text{-}P(4200)}=2.97$, $p_{\mathrm{PANAS}\text{-}P}=0.021$, ${\eta^{2}}_{\mathrm{PANAS}\text{-}N}=0.023$; $F_{\mathrm{PANAS}\text{-}N(4200)}=11.63$, $p_{\mathrm{PANAS}\text{-}N}<0.001$, ${\eta^{2}}_{\mathrm{PANAS}\text{-}N}=0.079$), but no main effect of group and no other interaction effects. These findings suggested that there were no unspecific effects of the training on mood that may interfere with cognitive improvement. Moreover, the training groups reported a comparable evaluation of their perceived training success (t=1.399, p=0.168, Cohen’s d=0.388) and experimental conditions (t=1.059, p=0.295, Cohen’s d=0.294).

**Table 1:** Demographic information and pretraining psychopathological symptom load in the two training groups, mean, and SDs (in brackets) are reported.

	Experimental group N=27 (8 males)	Control group N=25 (6 males)	Independent samples t-test
SAI	38.82(7.30)	40.28(8.47)	t=−0.670; p=0.506; and Cohen’s d=−0.186
TAI	40.00(7.40)	42.08(8.97)	t=−0.915; p=0.365; and Cohen’s d=−0.254
BDI II	5.37(5.82)	7.04(5.72)	t=−1.042; p=0.303; and Cohen’s d=−0.289
Age	21(1.99)	22(2.27)	/

SAI, state anxiety inventory; TAI, trait anxiety inventory; and BDI II, Beck depression inventory-II.

### 3.3 Primary Behavioral Outcomes

**Fig. 3:**
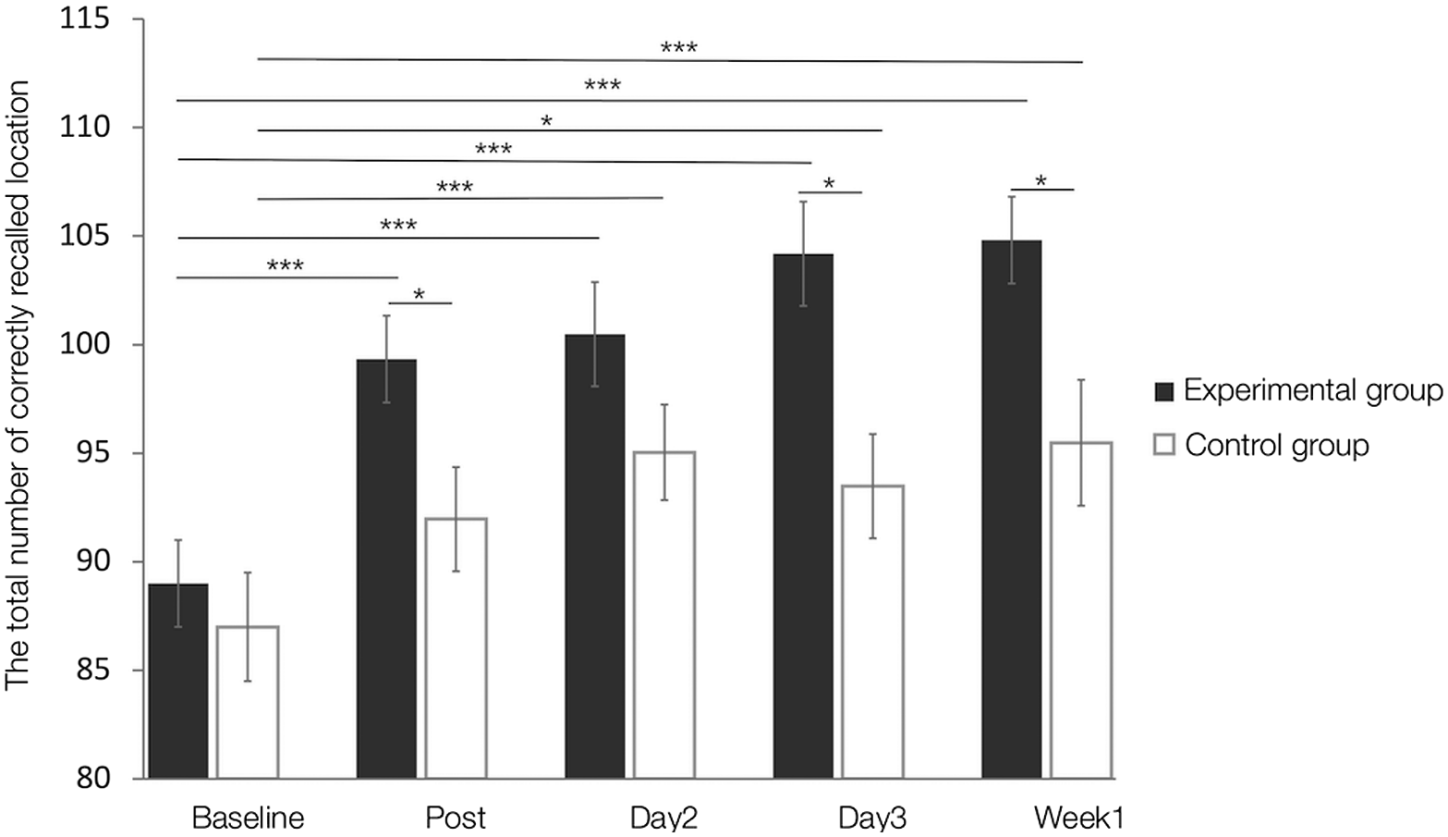
Changes in SWM forward task performance across all sessions. ∗p<0.05; ∗∗∗p<0.001. Error bars indicate the standard error of the mean (SEM).

Examining effects on the total number of correctly recalled locations in the forward tasks using mixed two-way ANOVA with the factors group (real feedback versus sham feedback) and time (baseline/post/day2/day3/week1) revealed a main effect of time ($F_{\left(4200\right)}=25.40$, p<0.001, $\eta^{2}=0.108$) and group ($F_{\left(150\right)}=6.16$, p=0.016, $\eta^{2}=0.073$), as well as a significant interaction effect between group and time ($F_{\left(4200\right)}=2.49$, p=0.045, $\eta^{2}=0.011$, see Fig. 3). Post-hoc comparisons demonstrated that the SWM performance significantly increased after the real feedback training (baseline < post, p<0.001; baseline < day2, p<0.001; baseline < day3, p<0.001; baseline < week1, p<0.001, two-tailed, Bonferroni-corrected, see Fig. 3). Concordant analysis of the sham group data also found the significant changes (baseline < day2, p<0.001; baseline < day3, p=0.003; baseline < week1, p<0.001, two-tailed, Bonferroni-corrected, see Fig. 3), which may be explained by the day-to-day practice effect. Importantly, directly comparing the training groups further revealed that the experimental group exhibited significantly better SWM performance during post/day3/week1-training sessions (ps < 0.031, two-tailed, Bonferroni-corrected, see Fig. 3) as compared to the control group; however, the group did not exhibit differences during baseline and day2-training sessions (ps > 0.108, two-tailed, Bonferroni-corrected), thus further confirming training success on the behavior level. However, the indices of the backward task revealed a main effect of time ($F_{\left(2100\right)}=36.16$, p<0.001, $\eta^{2}=0.087$) in the absence of the main effect of Group and the interaction effect (both p values>0.5).

**Fig. 4:**
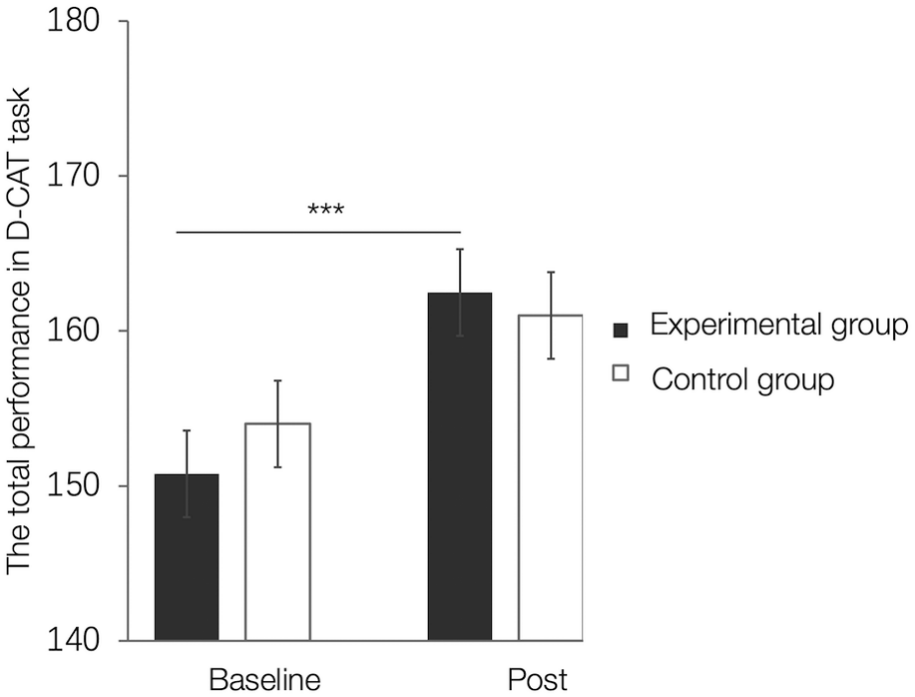
NF-induced behavioral changes in D-CAT task (Post versus baseline-training sessions).∗∗∗p<0.001. Error bars indicate the SEM.

Examining effects on the total performance in the D-CAT using mixed two-way ANOVA analyses with the factors group (real feedback versus sham feedback) and Time (baseline versus post) revealed the main effect of time ($F_{\left(150\right)}=22.61$, p<0.001, $\eta^{2}=0.023$) and the significant interaction effect between group and time ($F_{\left(150\right)}=4.29$, p=0.043, $\eta^{2}=0.004$, see Fig. 4) in the absence of the main effect of Group. Post-hoc comparisons demonstrated that performance significantly increased after the real feedback training (baseline < post, p<0.001, two-tailed). Concordant analysis of the sham training data did not yield significant results. However, a direct comparison between the groups did not reveal better performance in the experimental group as compared to the control group at baseline and post-training sessions (ps > 0.071, two-tailed).

### 3.4 Evaluation of Neural Training Success

**Fig. 5:**
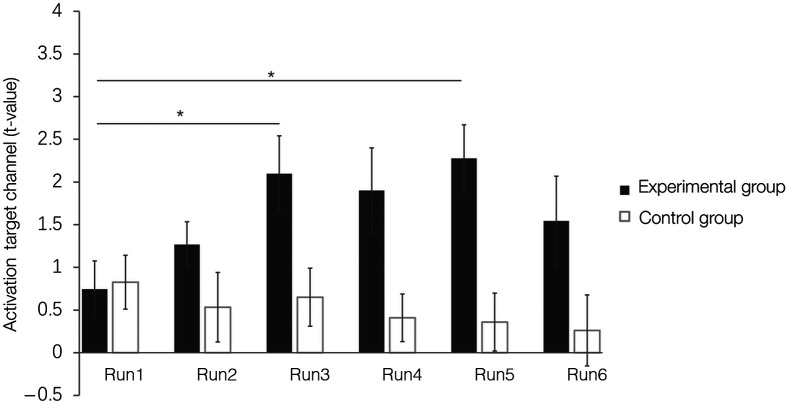
The oxy-Hb signal in the target channel significantly increased throughout the real feedback training runs but not during the sham NF training, *p<0.05. Error bars indicate the SEM.

A mixed two-way ANOVA with the factors time (run2/run3/run4/run5/run6) and group (real feedback versus sham feedback) and the dependent variable DLPFC activity as measured by the beta values (oxy-Hb) from the target channel revealed a main effect of group ($F_{\left(150\right)}=10.44$, p=0.002, $\eta^{2}=0.103$, see Fig. 5), but no main effect of Time and no other interaction effects. A one-way ANOVA with the factors Time (run1/run2/run3/run4/run5/run6) and the dependent variable DLPFC activity as measured by the beta values (oxy-Hb) from the target channel in the experimental group revealed a main effect of time ($F_{\left(5130\right)}=3.96$, p=0.002, $\eta^{2}=0.132$), post-hoc comparisons demonstrated that activation in the target channel significantly increased throughout the real feedback training (run1 < run3, p=0.045; run1 < run5, p=0.004, two-tailed, Bonferroni-corrected, see Fig. 5). Concordant analysis of the sham training data did not yield significant changes in the target channel ($F_{\left(5120\right)}=0.488$, p=0.785, $\eta^{2}=0.02$), thus further confirming training success on the neural level.

### 3.5 Association Between Behavioral Outcomes and Neural Training Success

**Fig. 6:**
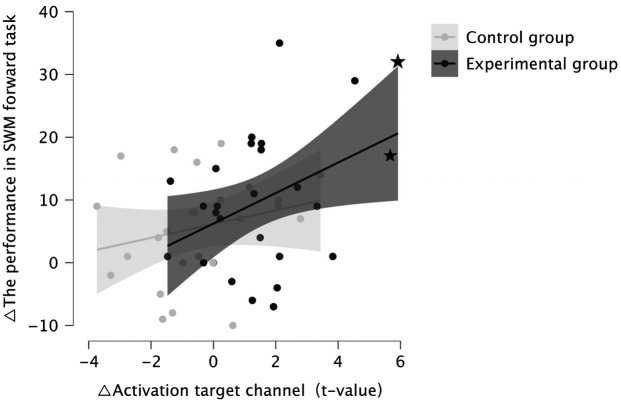
In the experimental group, stronger training-induced DLPFC activity changes (run5 > run1) were positively associated with better performance in the SWM forward task, and in the sham NF group, no association is observed. Outliers were marked by stars.

Given that previous studies have reported significant associations between fNIRS-assessed DLPFC activity and SWM,^33^^,^^35^^–^^38^ a subsequent correlation analysis examined the relationship between behavioral outcomes and neural training success. Results revealed that NF-induced activation changes in the DLPFC (changes in run5 > run1 activation in oxy-Hb in the target channel) were significantly and positively correlated with improved SWM performance (the difference in the total number of correctly recalled locations in the forward task between baseline and post-training sessions) in the experimental group (r=0.406, p=0.018; after excluding two outliers based on the in oxy-Hb, which showed higher neural learning success, r=0.232, p=0.132 one-tailed, see Fig. 6). Concordant analysis of the sham training data did not yield significant results: NF-induced activation changes in DLPFC were not correlated with changes in SWM performance (r=0.243, p=0.121, one-tailed, see Fig. 6).

To further confirm the association between training effects, a mediation analysis using bootstrap was conducted to explore the relationship between fNIRS-NF treatment (real feedback versus sham feedback) and the SWM performance changes.^59^ The simple mediation model can be formalized by the following equations:

M=i1+aX+e1, (1)

Y=i2+bM+cX+e2, (2)

$$Y=i3+c^{\prime }X+e3.$$ (3)

**Fig. 7:**
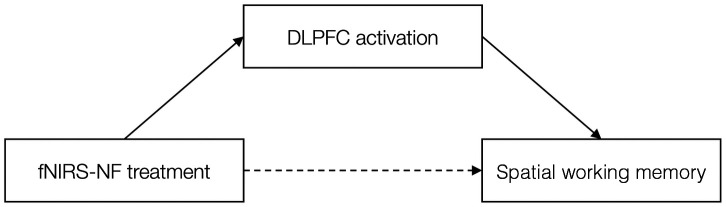
The mediation effect of the DLPFC activity on the relationship between the treatment and the SWM. The effect of fNIRS-NF treatment on SWM was fully mediated by the DLPFC activation in the experimental group.

This model reflects a causal sequence in which X (a predictor) is postulated to affect M (the mediator), and this effect then propagates causally to Y (the dependent variable). The previous study argued that there need not be a significant rXY in a proper mediation analysis and the only requirement for mediation is that the indirect effect X-M*M-Y be significant.^60^ The bootstrapping procedure a computer-intensive resampling technique first introduced by Efron (1979)^61^ has been proposed to test the significance of the indirect effect. The number of bootstrap samples for bias corrected bootstrap confidence intervals is 5000 and level of confidence for all confidence intervals is set to 95. Results revealed that the indirect effect ($\beta_{\text{Treatment}\text{-}\mathrm{DLPFC}}*\beta_{\mathrm{DLPFC}\text{-}\mathrm{SWM}}$) was significant (Effect = 0.355, BootSE = 0.167, BootCI = [0.098, 0.765]), suggesting fNIRS-NF treatment significantly increased the DLPFC activity and that increased DLPFC activity led to improved SWM performance, see Fig. 7 and Table 2.

**Table 2:** Mediation analysis of the relationship between the fNIRS-NF treatment and the SWM during the forward task among participants in the experimental group.

Effect	B	se	t (p-value)	CI	R2(ΔR2)	F
**Direct**						
Treatment → DLPFC	0.947	0.246	3.847(<0.001)	[0.453, 1.442]	0.478(0.228)	14.802
DLPFC → SWM	0.375	0.150	2.506(0.016)	[0.074, 0.675]	0.394(0.155)	4.490
Treatment → SWM	0.073	0.296	0.245(0.807)	[-0.523, 0.668]	0.032(0.001)	0.060
**Indirect**	Effect		Boot SE		BootCI	
Treatment → DLPFC → SWM $\beta_{\text{Treatment-}\mathrm{DLPFC}}*\beta_{\mathrm{DLPFC}\text{-}\mathrm{SWM}}$	0.355		0.167		[0.098, 0.765]	

In summary, these findings were consistent with other reports of training-driven modulations in prefrontal cortical activity, supporting training-induced neuroplasticity as the mechanistic basis of these behavioral training effects.

### 3.6 Exploratory Analysis – Regional Specificity of the Training Effects

These improved SWM and attention performance results suggested that a common, underlying mechanism of cognitive control was enhanced by fNIRS-NF. To assess this further, we examined the regional specificity of the fNIRS-NF training by quantifying the effect of training on all PFC channels. A mixed ANOVA with the factors Time (run2/run3/run4/run5/run6), group (real feedback versus sham feedback), channel (channels 1 to 22), and the dependent variable brain activity as measured by the oxy-Hb beta values revealed a main effect of group ($F_{(150)\;}=5.86$, p=0.019, $\eta^{2}=0.04$), a main effect of channel ($F_{(8390)\;}=4.05$, p<0.001, $\eta^{2}=0.016$), and a channel × group interaction effect ($F_{(8390)\;}=2.03$, p=0.043, $\eta^{2}=0.008$), but no main effect of time and no other interaction effects. To control for multiple comparisons, a Bonferroni correction was used to account for all channels tested. Post-hoc comparisons demonstrated that significant differences between the experimental group and the control group were observed in the target channel 9 (p=0.002, two-tailed) and the adjacent channels 3-8, 13, and 17-18 (all ps < 0.04, two-tailed). For all other channels, the interaction effect was not significant (all ps > 0.055, two-tailed).

A one-way ANOVA with the factors time (run1/run2/run3/run4/run5/run6) and the dependent variable DLPFC activity as measured by the beta values (oxy-Hb) in the experimental group revealed a main effect of time in channels 9 and 13 (channel 9: $F_{\left(5130\right)}=3.96$, p=0.002, $\eta^{2}=0.132$; channel 13: $F_{\left(5130\right)}=2.33$, p=0.046, $\eta^{2}=0.082$), post-hoc comparisons demonstrated that activation in the channels 9 and 13 significantly increased throughout the real feedback training (channel 9: run1< run3, p=0.045, run1 < run5, p=0.004; channel 13: run1 < run5, p=0.055, two-tailed, Bonferroni-correction). Concordant analysis of other adjacent channels did not yield significant changes (all ps>0.093), indicating that training specifically modulated activity in the right DLPFC (channels 9 and 13).

The effect of training on all TPJ channels was additionally explored. A mixed ANOVA with the factors time (run2/run3/run4/run5/run6), group (real feedback versus sham feedback), and channel (channels 1 to 24) and the dependent variable brain activity as measured by the oxy-Hb beta values revealed a main effect of channel ($F_{\left(8400\right)}=3.24$, p=0.001, $\eta^{2}=0.015$), but no other main effects and other interaction effects.

### 3.7 Exploratory Analysis – Training Effect on the Frontoparietal Connectivity

In addition, we analyzed the correlation between frontal and posterior brain regions, a functional connectivity measure also associated with cognitive control.^7^^,^^62^^–^^66^ Region-averaged oxy-Hb signals corresponding to the prefrontal cortex ($X_{A}$, averaged oxy-Hb signal from channels 9 and 13 in BA46, as shown in Table S1 in the Supplementary Material) and parietal cortex ($X_{B}$, averaged oxy-Hb signal from channels 11-12, 15-16, 18-24 in BA7, BA39, and BA40, as shown in Table S2 in the Supplementary Material) were calculated, and the Pearson’s correlation (r) between $X_{A}$ and $X_{B}$ was then calculated, and finally, a Fisher r-z transform was performed on the calculated Pearson’s correlation.^30^

The group difference of NF-induced frontoparietal connectivity was not significant in run1 ($t_{\left(50\right)}=0.73$, p=0.469, Cohen’s d=0.203, two-tailed). Then a mixed ANOVA with the factors time (run2/run3/run4/run5/run6), group (real feedback versus sham feedback), and the dependent variable frontoparietal connectivity as measured by the oxy-Hb z values revealed a main effect of group ($F_{\left(150\right)}=4.54$, p=0.038, $\eta^{2}=0.04$) in the absence of the main effect of time and the interaction effect, indicating that fNIRS-NF training of DLPFC modulated the frontoparietal connectivity.

### 3.8 Regulation Strategies Reported by the Participants

In line with a previous study that evaluated regulation strategies during NF training with the present platform,^58^ the content analysis identified five main clusters of upregulation strategies: (1) imagination, (2) experience recall, (3) concentration, (4) calculating, and (5) singing without a voice. Importantly, the groups did not differ in the regulation strategies employed during the training (Pearson χ2 test, p=0.856, two-tailed, Table 3), arguing against the confounding effects of different regulation strategies on the observed neural and behavioral between-group differences.

**Table 3:** Regulation strategies reported by the participants.

	Experimental group	Control group
Imagination	7	10
Experience recall	11	11
Concentration	2	1
Calculating	8	6
Singing without a voice	2	3

Numbers correspond to the number of participants reporting the corresponding regulation strategy in each training group.

## 4 Discussion

This present randomized sham-controlled between-subject real-time fNIRS design provides evidence for the feasibility of an fNIRS-informed NF training that enables individuals to gain control over right DLPFC activation. On the behavioral level, the training improved SWM performance, and this enhancement was maintained over several days. On the network level, the regional-specific enhancement in DLPFC activation induced changes in the network level, i.e., it modulated frontoparietal coupling. These results may point to a novel closed-loop strategy to induce a lasting improvement in SWM performance and to gain control over brain activity.

More specifically, the present study revealed that participants in the experimental group successfully learned to increase right DLPFC activity over six subsequent fNIRS-NF training runs. Importantly, no significant changes in neural activation were observed in the control group. Together with the lack of between-group differences in perceived training success and self-reported experimental conditions as well as regulation strategies, our results emphasized the specific importance of the feedback signal for the successful acquisition of neural regulation. Significant training-induced changes were restricted to the target (channel 9) and an adjacent channel (channel 13) suggesting that the training produced regional-specific increases in right DLPFC activation. Furthermore, the exploratory analysis revealed a significant group difference in NF-induced frontoparietal connectivity suggesting that the training may induce changes in the communication between core nodes of the SWM network. On the behavioral level, enhanced SWM performance in the forward task and improved attention performance in the D-CAT following the training of the fNIRS-NF were observed, importantly, the experimental group also demonstrated better SWM performance over the follow-up period for 2 days and 1 week. Moreover, exploratory analyses revealed that better SWM performance was associated with stronger training-induced increases in right DLPFC activity and increased DLPFC activity mediated the effects of fNIRS-NF treatment on better SWM performance providing evidence that the training-induced neural training effects critically contributed to the cognitive enhancement following training.

Comparing the experimental group with the control group demonstrated that fNIRS-NF allowed participants to acquire regulatory control over regional-specific activation in the right DLPFC. Examining the activation changes within the groups further documented that right DLPFC activity significantly increased over the six training runs in the experimental but not in the control group. Consistent with but extending past research,^24^ the present results suggested that fNIRS-NF training allows participants to gain volitional control over frontal cortical brain activity. DLPFC alterations have been demonstrated in several disorders characterized by deficits in cognitive function, particularly hypoactivity within this cortical region was observed in cognitive control among patients with schizophrenia,^63^ depression,^67^^–^^69^ and anxiety.^70^^,^^71^ Moreover, the exploratory analysis revealed that fNIRS-NF of DLPFC induced significant group differences in frontoparietal connectivity. Therefore, our results suggesting fNIRS-assisted regulatory control over DLPFC activity and frontoparietal connectivity may promote the normalization of aberrant neural activation and promote functional recovery in individuals with functional decline and psychiatric populations.

The DLPFC critically contributes to WM, with this region being considered to support the strategic control of working memory processing.^72^ In the present study, regional-specific modulation of the right DLPFC affected working memory performance in response to spatial stimuli, and the correlational analysis revealed that stronger training-induced DLPFC increases in the experimental group were associated with better SWM performance. Noteworthy, excluding two participants with the highest learning success changed the significance but not the direction of the effects. This may suggest that individual variations strongly affect NF training success which has been an ongoing debate in the NF field. A number of recent large-scale studies have investigated factors that may contribute to individual differences in learning success.^73^^–^^75^ Furthermore, a mediation analysis demonstrated that increased DLPFC activation mediated the effects of fNIRS-NF treatment on better SWM performance. Previous studies demonstrated that patients with frontal-lobe damage are impaired by the inefficient use of organizational strategies that improve performance in healthy controls,^72^ which may explain the strong performance effects on the SWM paradigm in the present study. Notably repeated administration of SWM also lead to improved performance in the control group, suggesting a practice effect for the behavioral task. However, the experimental group performed better during post/day3/week1-training sessions compared to the control group, suggesting that the practice effect alone cannot explain the improved performance of the experimental group. Moreover, the experimental group demonstrated a significant improvement in SWM performance that lasted for one week relative to the control group. The maintenance of the training effects on the behavioral level is critical for the translation into daily life and novel treatments. Interestingly, the performance enhancement was more significant on the third day after training, which may point to the role of sleep in learning consolidation.^76^ Although the present study only employed a single training session, the maintenance of training-induced behavioral effects may suggest that fNIRS-NF may represent a promising and scalable intervention to induce long-term effective improvement for SWM.

The present findings need to be interpreted in the context of limitations. First, the present study was conducted with a healthy and sex-biased sample. The potential sex differences and generalization of the present results to patient populations need to be examined in future studies. Second, despite some evidence for the effects of fNIRS-NF training of right DLPFC on SWM performance, the between-group comparisons of the indices of the backward task failed to reach statistical significance. The lack of robust effects in this domain may be explained in terms of forward and backward recall calling upon qualitatively distinct retrieval processes.^77^ Previous studies have revealed that the visuospatial span backward task was more difficult and involved greater WM demands than the forward, especially, the backward task may be associated with greater participation of the central executive as an attentional control system.^44^^,^^45^ Consistent with the previous study, our results may reflect that the forward task mainly relies on updating data while the backward task additionally measures a manipulation of the information in the storage. In line with the main aim of the study, the primary assessments of the maintenance effect focused on SWM while the attention test was administered only once. Future studies are needed to examine long-term effects on other cognitive domains, such as attention. Third, the present study focused on training effects on the behavioral level and did not include neural assessments after the NF training. So, the underlying neural mechanisms of NF-induced better SWM performance remain unclear. A recent study presented a WM challenge after fNIRS-informed DLPFC training with concomitant fMRI and reported that the training enhanced DLPFC activation during WM in the absence of feedback.^78^ Future studies may therefore acquire fNIRS or fMRI during the maintenance sessions to determine which neural correlates mediate the maintenance of the enhanced SWM performance during follow-up sessions. In addition, the present study attempted to reveal the neural changes related to NF-induced better SWM performance in terms of functional connectivity. Based on the activation results the analysis specifically focused on the connectivity of channels that exhibited significant training effects. Future studies should investigate more complex connectivity measures including all channels. Fourth, plotting activation of the target channel over the training runs revealed that learning occurred after the two initial runs with no further increase from run 3 to run 6. This indicates that learning during the training might not have followed a linear increase over the six runs but rather regulation ability changed qualitatively after the third run. Notably, a similar learning curve has been observed in previous fMRI NF studies.^41^ Some studies indicated this might be due to ceiling effects,^23^ while others focusing on learning curves of NF revealed that subjects were not able to remain motivated towards the end of the session or that they could not sustain the energy demands of the training.^79^ In particular, they interpret these results as time-on-task effects; it seems that the participants grew tired after some time and could not sustain attention.^80^ Although the NF training was less instructive and was perceived as relaxing, this may still have influenced the participant’s ability to regulate brain activity. Taken together, these aspects could explain the lack of a systematic learning effect throughout the training. In this regard, to substantiate participants’ ability to upregulate prefrontal activity, future studies should include transfer trials in which participants have to upregulate prefrontal activity without any direct feedback of their HR.^41^ The absence of feedback during regulation trials can support a conclusion on how good a person got a feel for the actual brain state and how to modulate it.^23^ Finally, in the majority of prior NIRS-based NF studies participants were instructed to up-regulate the NIRS signal.^81^ In contrast, studies examining the ability to decrease or down-regulate the NIRS signal are scarce. Hosseini et al. (2016) found mixed effects on working memory after task-based NF training targeting the down-regulation of the DLPFC.^24^ Performance improved in an n-back task as compared to the sham-feedback group, but no improvement was found in either group for the delayed verbal working-memory task used for the task-based NF paradigm. There is currently no consensus on whether training to up-regulate or down-regulate activity in a target region is more efficient to improve working memory or other cognitive functions^22^ and an optimal trial structure for fNIRS-NF to improve executive function procedures needs to be determined in future studies. The combination of up-and down-regulation in one training session might for instance be a promising idea for future studies.

In summary, the present findings demonstrated that real-time fNIRS-NF training allows healthy individuals to gain control over DLPFC activity and that this leads to improved SWM performance, which is maintained for at least 1 week after the training and in the absence of further online feedback. Both the behavioral and neural results of this study support the hypothesis that the right DLPFC plays a crucial role in SWM performance and may specifically support attentional control. We provide promising results with the therapeutic potential of fNIRS-NF to target SWM and its underlying neural basis, which are impaired in both aging populations and a range of psychiatric disorders.

## Code, Data, and Materials Availability

The data that support the findings of this study are available from the corresponding author upon reasonable request.

## References

[1] Baddeley A., “Working memory: looking back and looking forward,”Nat. Rev. Neurosci. 4(10), 829–839 (2003). NRNAAN 1471-003X 10.1038/nrn1201 14523382

[2] Jaroslawska A. J. Rhodes S., “Adult age differences in the effects of processing on storage in working memory: a meta-analysis,” Psychol. Aging 34(4), 512–530 (2019). 10.1037/pag0000358 31045391

[3] Cho Y. T. et al., “Effects of reward on spatial working memory in schizophrenia,” J. Abnormal Psychol. 127(7), 695–709 (2018). JAPCAC 0021-843X 10.1037/abn0000369 PMC6197071 30335439

[4] Muster R. et al., “Mapping the neuroanatomic substrates of cognition in familial attention deficit hyperactivity disorder,” Psychol. Med. 49(4), 590–597 (2019). PSMDCO 0033-2917 10.1017/S0033291718001241 29792238 PMC6252155

[5] Martinussen R. et al., “A meta-analysis of working memory impairments in children with attention-deficit/hyperactivity disorder,” J. Am. Acad. Child Adolesc. Psychiatry 44(4), 377–384 (2005). JAAPEE 0890-8567 10.1097/01.chi.0000153228.72591.73 15782085

[6] Semkovska M. et al., “Cognitive function following a major depressive episode: a systematic review and meta-analysis,” Lancet Psychiatry 6(10), 851–861 (2019). 10.1016/S2215-0366(19)30291-3 31422920

[7] Wagner S. et al., “A meta-analysis of cognitive functions in children and adolescents with major depressive disorder,” Eur. Child Adolesc. Psychiatry 24, 5–19 (2015). EAPSE9 1435-165X 10.1007/s00787-014-0559-2 24869711

[8] Tian F. et al., “Prefrontal responses to digit span memory phases in patients with post-traumatic stress disorder (PTSD): a functional near infrared spectroscopy study,” Neuroimage Clin. 4, 808–819 (2014). 10.1016/j.nicl.2014.05.005 24936431 PMC4055895

[9] Vance A. et al., “Examination of spatial working memory performance in children and adolescents with attention deficit hyperactivity disorder, combined type (ADHD-CT) and Anxiety,” J. Abnormal Child Psychol. 41(6), 891–900 (2013). JABCAA 0091-0627 10.1007/s10802-013-9721-4 23378043

[10] Awh E. Jonides J., “Overlapping mechanisms of attention and spatial working memory,” Trends Cognit. Sci. 5, 119–126 (2001). TCSCFK 1364-6613 10.1016/S1364-6613(00)01593-X 11239812

[11] Segen V. et al., “Age-related differences in visual encoding and response strategies contribute to spatial memory deficits,” Mem. Cognit. 49(2), 249–264 (2021). MYCGAO 0090-502X 10.3758/s13421-020-01089-3 PMC7886755 32869141

[12] Kerchner G. A. et al., “Cognitive processing speed in older adults: relationship with white matter integrity,” PLoS ONE 7(11), e50425 (2012). POLNCL 1932-6203 10.1371/journal.pone.0050425 23185621 PMC3503892

[13] Moore S. C. Oaksford M., “Some long-term effects of emotion on cognition,” Br. J. Psychol. 93(3), 383–395 (2002) BJSGAE 0007-1269 10.1348/000712602760146341 12230836

[r14] Smallwood J. O’Connor R. C., “Imprisoned by the past: unhappy moods lead to a retrospective bias to mind wandering,” Cognit. Emotion 25(8), 1481–1490 (2011). COEMEC 10.1080/02699931.2010.545263 21432633

[r15] Lavric A. Rippon G. Gray J. R., “Threat-evoked anxiety disrupts spatial working memory performance: an attentional account,” Cognit. Ther. Res. 27(5), 489–504 (2003). CTHRD8 0147-5916 10.1023/A:1026300619569

[r16] Gerrits R. et al., “Mirrored brain organization: statistical anomaly or reversal of hemispheric functional segregation bias?,” Proc. Natl. Acad. Sci. U. S. A. 117(25), 14057–14065 (2020). 10.1073/pnas.2002981117 32513702 PMC7322015

[17] Shackman A. J. et al., “Anxiety selectively disrupts visuospatial working memory,” Emotion 6, 40–61 (2006). EBMOEN 10.1037/1528-3542.6.1.40 16637749

[18] Birbaumer N. Ruiz S. Sitaram R., “Learned regulation of brain metabolism,” Trends Cognit. Sci. 17, 295–302 (2013). TCSCFK 1364-6613 10.1016/j.tics.2013.04.009 23664452

[19] Thibault R. T. et al., “Neurofeedback, self-regulation, and brain imaging: clinical science and fad in the service of mental disorders,” Psychother. Psychosom. 84(4), 193–207 (2015). PSPSBF 0033-3190 10.1159/000371714 26021883

[20] Watanabe T. et al., “Advances in fMRI real-time neurofeedback,” Trends Cognit. Sci. 21, 997–1010 (2017). TCSCFK 1364-6613 10.1016/j.tics.2017.09.010 29031663 PMC5694350

[21] Ferrari M. Quaresima V., “A brief review on the history of human functional near-infrared spectroscopy (fNIRS) development and fields of application,” NeuroImage 63, 921–935 (2012). NEIMEF 1053-8119 10.1016/j.neuroimage.2012.03.049 22510258

[22] Kohl S. H. et al., “The potential of functional near-infrared spectroscopy-based neurofeedback—a systematic review and recommendations for best practice,” Front. Neurosci. 14, 594 (2020).1662-453X 10.3389/fnins.2020.00594 32848528 PMC7396619

[23] Barth B. et al., “Near-infrared spectroscopy based neurofeedback of prefrontal cortex activity: a proof-of-concept study,” Front. Hum. Neurosci. 10, 633 (2016). 10.3389/fnhum.2016.00633 28018199 PMC5159415

[24] Hosseini S. M. H. et al., “Task-based neurofeedback training: a novel approach toward training executive functions,” NeuroImage 134, 153–159 (2016). NEIMEF 1053-8119 10.1016/j.neuroimage.2016.03.035 27015711 PMC4912915

[25] Li K. et al., “Functional near-infrared spectroscopy-informed neurofeedback: regional-specific modulation of lateral orbitofrontal activation and cognitive flexibility,” Neurophotonics 6(2), 025011 (2019). 10.1117/1.NPh.6.2.025011 31930153 PMC6951484

[26] Hou X. et al., “Functional near-infrared spectroscopy neurofeedback enhances human spatial memory,” Front. Hum. Neurosci. 15, 681193 (2021) 10.3389/fnhum.2021.681193 34658812 PMC8511425

[27] Marx A. M. et al., “Near-infrared spectroscopy (NIRs) neurofeedback as a treatment for children with attention deficit hyperactivity disorder (ADHD)—a pilot study,” Front. Hum. Neurosci. 8, 1038 (2015). 10.3389/fnhum.2014.01038 25610390 PMC4285751

[r28] Mihara M. et al., “Near-infrared spectroscopy-mediated neurofeedback enhances efficacy of motor imagery-based training in poststroke victims: a pilot study,” Stroke 44(4), 1091–1098 (2013). SJCCA7 0039-2499 10.1161/STROKEAHA.111.674507 23404723

[r29] Narita N., “Application of NIRS as a non-invasive and supportive tool for autism spectrum disorders,” Trans. Jpn. Soc. Med. Biol. Eng. 53, S153_01 (2015). 10.11239/jsmbe.53.S153_01

[30] Xia M. et al., “Frontoparietal connectivity neurofeedback training for promotion of working memory: an fNIRS study in healthy male participants,” IEEE Access 9, 62316–62331 (2021). 10.1109/ACCESS.2021.3074220

[31] Crone E. A. Steinbeis N., “Neural perspectives on cognitive control development during childhood and adolescence,” Trends Cognit. Sci. 21, 205–215 (2017). TCSCFK 1364-6613 10.1016/j.tics.2017.01.003 28159355

[32] Zwanzger P. et al., “Inhibitory repetitive transcranial magnetic stimulation (rTMS) of the dorsolateral prefrontal cortex modulates early affective processing,” NeuroImage 101, 193–203 (2014). NEIMEF 1053-8119 10.1016/j.neuroimage.2014.07.003 25019678

[33] Wu Y. J. et al., “Modulating the interference effect on spatial working memory by applying transcranial direct current stimulation over the right dorsolateral prefrontal cortex,” Brain Cognit. 91, 87–94 (2014). 10.1016/j.bandc.2014.09.002 25265321

[34] Sauseng P. et al., “Brain oscillatory substrates of visual short-term memory capacity,” Curr. Biol. 19(21), 1846–1852 (2009). CUBLE2 0960-9822 10.1016/j.cub.2009.08.062 19913428

[35] Kronovsek T. et al., “Age-related decline in visuo-spatial working memory is reflected by dorsolateral prefrontal activation and cognitive capabilities,” Behav. Brain Res. 398, 112981 (2021) BBREDI 0166-4328 10.1016/j.bbr.2020.112981 33144176

[r36] Luna B. et al., “Neocortical system abnormalities in autism: an fMRI study of spatial working memory,” Neurology 59(6), 834–840 (2002). NEURAI 0028-3878 10.1212/WNL.59.6.834 12297562

[r37] Silk T. J. et al., “Visuospatial processing and the function of prefrontal-parietal networks in autism spectrum disorders: a functional MRI study,” Am. J. Psychiatry 163(8), 1440–1443 (2006). 10.1176/ajp.2006.163.8.1440 16877661

[38] Gamo N. J. Arnsten A. F. T., “Molecular modulation of prefrontal cortex: rational development of treatments for psychiatric disorders,” Behav. Neurosci. 125(3), 282–296 (2011). BENEDJ 0735-7044 10.1037/a0023165 21480691 PMC3109197

[39] Herff C. et al., “Mental workload during n-back task-quantified in the prefrontal cortex using fNIRS,” Front. Hum. Neurosci. 7, 935 (2014). 10.3389/fnhum.2013.00935 24474913 PMC3893598

[40] Yao S. et al., “Voluntary control of anterior insula and its functional connections is feedback-independent and increases pain empathy,” NeuroImage 130, 230–240 (2016). NEIMEF 1053-8119 10.1016/j.neuroimage.2016.02.035 26899786

[41] Zhao Z. et al., “Real-time functional connectivity-informed neurofeedback of amygdala-frontal pathways reduces anxiety,” Psychother. Psychosom. 88(1), 5–15 (2019). PSPSBF 0033-3190 10.1159/000496057 30699438

[42] Thibault R. T. Pedder H., “Excess significance and power miscalculations in neurofeedback research,” Neuroimage Clin. 35, 103008 (2022). 10.1016/j.nicl.2022.103008 35525708 PMC9421468

[43] Salmi J. et al., “Working memory training restores aberrant brain activity in adult attention-deficit hyperactivity disorder,” Hum. Brain Mapp. 41(17), 4876–4891 (2020). 10.1002/hbm.25164 32813290 PMC7643386

[44] Wilde N. Strauss E., “Functional equivalence of WAIS-III/WMS-III digit and Spatial Span under forward and backward recall conditions,” Clin. Neuropsychol. 16(3), 322–330 (2002). 10.1076/clin.16.3.322.13858 12607145

[45] Guevara M. A. et al., “EEG activity during the spatial span task in young men: differences between short-term and working memory,” Brain Res. 1683, 86–94 (2018). BRREAP 0006-8993 10.1016/j.brainres.2018.02.004 29425909

[46] Hatta T. et al., “Reliability and validity of the digit cancellation test, a brief screen of attention,” Psychologia 55(4), 246–256 (2012). PYLGAY 10.2117/psysoc.2012.246

[47] Spielberger C. D. et al., “The state-trait anxiety inventory,” Rev. Int. Psicol./Interam. J. Psychol. 5, 3–4 (1971).

[48] Beck A. T. Steer R. A. Brown G. K., Manual for the Beck Depression Inventory-II, pp. 1–82, Psychological Corporation, San Antonio, Texas (1996)

[49] Watson D. Clark L. A. Tellegen A., “Development and validation of brief measures of positive and negative affect: the PANAS scales,” J. Person. Soc. Psychol. 54(6), 1063–1070 (1988). JPSPB2 0022-3514 10.1037/0022-3514.54.6.1063 3397865

[50] Reindl V. et al., “Brain-to-brain synchrony in parent-child dyads and the relationship with emotion regulation revealed by fNIRS-based hyperscanning,” NeuroImage 178, 493–502 (2018). NEIMEF 1053-8119 10.1016/j.neuroimage.2018.05.060 29807152

[51] Liu J. et al., “Interplay between prior knowledge and communication mode on teaching effectiveness: interpersonal neural synchronization as a neural marker,” NeuroImage 193, 93–102 (2019). NEIMEF 1053-8119 10.1016/j.neuroimage.2019.03.004 30851445

[52] Lapborisuth P. et al., “Neurofeedback-based functional near-infrared spectroscopy upregulates motor cortex activity in imagined motor tasks,” Neurophotonics 4(2), 021107 (2017). 10.1117/1.NPh.4.2.021107 28680906 PMC5482291

[53] Holtmann M. et al., “Near-infrared spectroscopy (NIRS) neurofeedback as a treatment for children with attention deficit hyperactivity disorder (ADHD)—a pilot study,” Front. Hum. Neurosci. 8, 1038 (2015). 10.3389/fnhum.2014.01038 25610390 PMC4285751

[54] Thibault R. T., “Neurofeedback with fMRI: a critical systematic review,” NeuroImage 172, 786–807 (2018). NEIMEF 1053-8119 10.1016/j.neuroimage.2017.12.071 29288868

[55] Ye J. C. et al., “NIRS-SPM: statistical parametric mapping for near-infrared spectroscopy,” NeuroImage 44(2), 428–447 (2009). NEIMEF 1053-8119 10.1016/j.neuroimage.2008.08.036 18848897

[56] Friston K., “Statistical parametric mapping,” 1991, https://www.fil.ion.ucl.ac.uk/spm/.

[57] Huppert T. J., “Commentary on the statistical properties of noise and its implication on general linear models in functional near-infrared spectroscopy,” Neurophotonics 3(1), 010401 (2016). 10.1117/1.NPh.3.1.010401 26989756 PMC4773699

[58] Kirsch M. et al., “Real-time functional magnetic resonance imaging neurofeedback can reduce striatal cue-reactivity to alcohol stimuli,” Addict. Biol. 21(4), 982–992 (2016). 10.1111/adb.12278 26096546

[59] Alfons A. Ateş N. Y. Groenen P. J. F., “A robust bootstrap test for mediation analysis,” Organ. Res. Methods 25(3), 591–617 (2022).1094-4281 10.1177/1094428121999096

[60] Zhao X. Lynch J. G. Jr. Chen Q., “Reconsidering Baron and Kenny: myths and truths about mediation analysis,” J. Consum. Res. 37(2), 197–206 (2010). 10.1086/651257

[61] Efron B., “Bootstrap methods: another look at the jackknife,” Ann. Stat. 7(1), 1–26 (1979).

[62] Jolles D. D. et al., “Functional brain connectivity at rest changes after working memory training,” Hum. Brain Mapp. 34(2), 396–406 (2013). 10.1002/hbm.21444 22076823 PMC6870317

[r63] Repovš G. Barch D. M., “Working memory related brain network connectivity in individuals with schizophrenia and their siblings,” Front. Hum. Neurosci. 6, 137 (2012). 10.3389/fnhum.2012.00137 22654746 PMC3358772

[r64] Sala-Llonch R. et al., “Brain connectivity during resting state and subsequent working memory task predicts behavioural performance,” Cortex 48(9), 1187–1196 (2012). 10.1016/j.cortex.2011.07.006 21872853

[r65] Cole M. W. et al., “Global connectivity of prefrontal cortex predicts cognitive control and intelligence,” J. Neurosci. 32(26), 8988–8999 (2012). JNRSDS 0270-6474 10.1523/JNEUROSCI.0536-12.2012 22745498 PMC3392686

[66] Sauseng P. et al., “Dissociation of sustained attention from central executive functions: local activity and interregional connectivity in the theta range,” Eur. J. Neurosci. 25(2), 587–593 (2007). EJONEI 0953-816X 10.1111/j.1460-9568.2006.05286.x 17284201

[67] Fales C. L. et al., “Altered emotional interference processing in affective and cognitive-control brain circuitry in major depression,” Biol. Psychiatry 63(4), 377–384 (2008). BIPCBF 0006-3223 10.1016/j.biopsych.2007.06.012 17719567 PMC2268639

[r68] Ebneabbasi A. et al., “Emotion processing and regulation in major depressive disorder: a 7T resting-state fMRI study,” Hum. Brain Mapp. 42(3), 797–810 (2021). 10.1002/hbm.25263 33151031 PMC7814754

[69] Erk S. et al., “Acute and sustained effects of cognitive emotion regulation in major depression,” J. Neurosci. 30(47), 15726–15734 (2010). JNRSDS 0270-6474 10.1523/JNEUROSCI.1856-10.2010 21106812 PMC6633759

[70] Bishop S. J., “Trait anxiety and impoverished prefrontal control of attention,” Nat. Neurosci. 12(1), 92–98 (2009). NANEFN 1097-6256 10.1038/nn.2242 19079249

[71] Moran T. P., “Anxiety and working memory capacity: a meta-analysis and narrative review,” Psychol. Bull. 142(8), 831–864 (2016). PSBUAI 0033-2909 10.1037/bul0000051 26963369

[72] Owen A. M. et al., “N-back working memory paradigm: a meta-analysis of normative functional neuroimaging studies,” Hum. Brain Mapp. 25(1), 46–59 (2005). 10.1002/hbm.20131 15846822 PMC6871745

[73] Haugg A. et al. “Predictors of real-time fMRI neurofeedback performance and improvement–a machine learning mega-analysis,” Neuroimage 237, 118207 (2021). NEIMEF 1053-8119 10.1016/j.neuroimage.2021.118207 34048901

[r74] Haugg A., et al. “Can we predict real‐time fMRI neurofeedback learning success from pretraining brain activity?,” Hum. Brain Mapp. 41(14), 3839–3854 (2020). 10.1002/hbm.25089 32729652 PMC7469782

[75] Zhao Z., et al. “Putamen volume predicts real‐time fMRI neurofeedback learning success across paradigms and neurofeedback target regions,” Hum. Brain Mapp. 42(6), 1879–1887 (2021). 10.1002/hbm.25336 33400306 PMC7978128

[76] Wang S. Y. et al., “Sleep-dependent’ memory consolidation? Brief periods of post-training rest and sleep provide an equivalent benefit for both declarative and procedural memory,” Learn. Mem. 28(6), 195–203 (2021). 10.1101/lm.053330.120 34011516 PMC8139635

[77] Guitard D. et al., “Forward and backward recall: different visuospatial processes when you know what’s coming,” Mem. Cognit. 48(1), 111–126 (2020). MYCGAO 0090-502X 10.3758/s13421-019-00966-w 31346926

[78] Yang X. et al., “A brief real-time fNIRS-informed neurofeedback training of the prefrontal cortex changes brain activity and connectivity during subsequent working memory challenge,” BioRxiv.org 2023.03.14.532684 (2023). 10.1016/j.pnpbp.2024.110968 38354898

[79] Janssen T. W.P., et al. “Learning curves of theta/beta neurofeedback in children with ADHD,” Europ. Child Adolesc. Psychiatry 26, 573–582 (2017). 10.1007/s00787-016-0920-8 PMC5394134 27866283

[80] Dekker M. K. J. et al. “The time-course of alpha neurofeedback training effects in healthy participants,” Biol. Psychol. 95, 70–73 (2014). BLPYAX 0301-0511 10.1016/j.biopsycho.2013.11.014 24321361

[81] Kober S. E. et al. “Trainability of hemodynamic parameters: a near-infrared spectroscopy based neurofeedback study,” Biol. Psychol. 136, 168–180 (2018). BLPYAX 0301-0511 10.1016/j.biopsycho.2018.05.009 29782968

