# Supplementary material for: Functional near-infrared spectroscopy neurofeedback of dorsolateral prefrontal cortex enhances human spatial working memory
Source: Neurophotonics. 2023 Jun 1;10(2):025011. doi: 10.1117/1.NPh.10.2.025011 (PMC10234406; doi:10.1117/1.NPh.10.2.025011)
Supplement: Supplementary file 1 [file NPh_010_025011_SD001.pdf]

Functional Near-Infrared Spectroscopy Neurofeedback of Dorsolateral Prefrontal Cortex Enhances Human Spatial Working Memory

Authors Li et al.
Contact keshuangli@outlook.com

Supplementary tables

Table S1. NIRS channels positions in PFC

Channel	MNI			AAL		Brodmann	
	x	y	z	Brain regions	Percentage	Brain regions	Percentage
1	-35.86	62.60	-7.25	Frontal_Mid_Orb_L	1	10 - Frontopolar area	0.58
2	-12.87	71.46	-3.23	Frontal_Mid_Orb_L	0.56	11 - Orbitofrontal area	0.77
3	15.02	71.07	-3.32	Frontal_Sup_Orb_R	0.83	11 - Orbitofrontal area	0.62
4	38.22	63.02	-7.03	Frontal_Mid_Orb_R	0.96	10 - Frontopolar area	0.62
5	-45.37	52.59	1.12	Frontal_Mid_L	0.52	46 - Dorsolateral prefrontal cortex	1
6	-24.37	67.88	8.48	Frontal_Sup_L	0.96	10 - Frontopolar area	0.97
7	2.13	67.98	9.10	Frontal_Sup_Medial_L	0.58	10 - Frontopolar area	1
8	26.88	67.98	8.45	Frontal_Sup_R	1	10 - Frontopolar area	0.93
9	46.73	53.15	1.72	Frontal_Mid_R	0.59	46 - Dorsolateral prefrontal cortex	1
10	-35.32	57.95	18.71	Frontal_Mid_L	1	46 - Dorsolateral prefrontal cortex	0.92
11	-12.73	66.80	21.62	Frontal_Sup_L	0.86	10 - Frontopolar area	1
12	14.49	67.95	22.79	Frontal_Sup_R	0.74	10 - Frontopolar area	1
13	37.66	58.54	18.50	Frontal_Mid_R	0.89	46 - Dorsolateral prefrontal cortex	0.79
14	-45.01	41.70	27.39	Frontal_Mid_L	0.89	45 - pars triangularis Broca's area	0.92
15	-23.41	56.21	32.67	Frontal_Sup_L	0.58	46 - Dorsolateral prefrontal cortex	0.65
16	1.51	59.22	34.10	Frontal_Sup_Medial_L	0.74	10 - Frontopolar area	0.53
17	25.75	57.02	32.92	Frontal_Sup_R	0.57	46 - Dorsolateral prefrontal cortex	0.50
18	47.08	41.58	27.84	Frontal_Mid_R	0.89	45 - pars triangularis Broca's area	0.94
19	-34.53	40.25	41.75	Frontal_Mid_L	0.92	9 - Dorsolateral prefrontal cortex	0.91
20	-11.21	50.38	45.42	Frontal_Sup_L	0.68	9 - Dorsolateral prefrontal cortex	1
21	13.13	50.43	45.72	Frontal_Sup_Medial_R	0.51	9 - Dorsolateral prefrontal cortex	1
22	36.16	39.80	42.14	Frontal_Mid_R	1	9 - Dorsolateral prefrontal cortex	0.97

Table S1. One 3*5 probe patch (3cm distance between the emitter and detector) was placed over the prefrontal regions of each participant. The correspondence between the fNIRS channels and the measurement points was referred to in the positioning template provided by Jichi University (http://www.jichi.ac.jp/brainlab/virtual_registration/Result3x5_E.html).

Table S2. NIRS channels positions in TPJ

Channel	MNI			AAL		Brodmann	
	x	y	z	Brain regions	Percentage	Brain regions	Percentage
1	65	-59	-6	Temporal_Inf_R	0.57	37 - Fusiform gyrus	0.98
2	52	-80	-5	Occipital_Inf_R	0.64	19 - V3	0.93
3	38	-96	-7	Occipital_Inf_R	0.79	18 - V2	0.91
4	69	-49	13	Temporal_Mid_R	0.70	22 - Superior Temporal Gyrus	0.56
5	58	-71	12	Temporal_Mid_R	0.91	37 - Fusiform gyrus	0.51
6	46	-88	9	Occipital_Mid_R	0.98	19 - V3	0.74
7	27	-103	6	Occipital_Sup_R	0.37	17 - V1	0.86
8	63	-60	25	Temporal_Mid_R	0.50	22 - Superior Temporal Gyrus	0.44
9	51	-79	24	Occipital_Mid_R	0.79	39 - Angular gyrus	0.64
10	34	-95	18	Occipital_Mid_R	0.69	18 - V2	0.52
11	64	-47	43	SupraMarginal_R	0.50	40 - Supramarginal gyrus	0.98
12	55	-67	41	Angular_R	0.88	39 - Angular gyrus	1
13	40	-83	38	Occipital_Mid_R	0.66	19 – V3	0.79
14	21	-96	30	Occipital_Sup_R	1	18 - V2	0.90
15	56	-56	51	Parietal_Inf_R	0.75	40 - Supramarginal gyrus	0.60
16	44	-74	48	Angular_R	0.84	39 - Angular gyrus	0.46
17	27	-86	45	Occipital_Sup_R	0.63	19 - V3	0.80
18	58	-42	56	Parietal_Inf_R	0.81	40 - Supramarginal gyrus	0.86
19	46	-62	57	Angular_R	0.42	40 - Supramarginal gyrus	0.37
20	28	-78	55	Parietal_Sup_R	0.86	7 - Somatosensory Association Cortex	0.93
21	16	-85	49	Cuneus_R	0.45	7 - Somatosensory Association Cortex	0.52
22	42	-49	65	Parietal_Sup_R	0.70	40 - Supramarginal gyrus	0.47
23	31	-64	67	Parietal_Sup_R	1	7 - Somatosensory Association Cortex	1
24	17	-76	62	Parietal_Sup_R	0.74	7 - Somatosensory Association Cortex	1

Table S2. One 4*4 probe patch was placed over the rTPJ of each participant. The correspondence between the fNIRS channels and the measurement points on the cerebral cortex was displayed based on the results of the virtual registration method, which had been confirmed by a multi-subject study of anatomical craniocerebral correlation[49].
